# New treatment options for inflammatory bowel diseases

**DOI:** 10.1007/s00535-018-1449-z

**Published:** 2018-03-19

**Authors:** Bram Verstockt, Marc Ferrante, Séverine Vermeire, Gert Van Assche

**Affiliations:** Division of Gastroenterology and Hepatology, University Hospitals Leuven and TARGID, University of Leuven, Herestaat 49, 3000 Louvain, Belgium

**Keywords:** IBD, Anti-IL23, Anti-adhesion, Jakinibs, FMT

## Abstract

The advent of anti-TNF agents has dramatically changed the treatment algorithms for IBD in the last 15 years, but primarily and more importantly secondary loss of response is often observed. Fortunately , new treatment options have been actively explored and some have already entered our clinical practice. In the class of anti-cytokine agents, the anti-IL12/IL23 monoclonal antibodies (mAbs) have entered clinical practice with the anti-p40 mAb ustekinumab in Crohn’s disease (CD). Also, more selective anti-IL23 agents (anti-p19) have shown efficacy and are being further developed, in contrast to agents inhibiting IL-17 downstream which have failed in clinical trials despite their clear efficacy in psoriasis (Verstockt et al. in Expert Opin Biol Ther 17(1):31–47, 2017; Verstockt et al. in Expert Opin Drug Saf 16(7):809–821, 2017). Following up on the efficacy of the anti-adhesion molecule vedolizumab, etrolizumab (anti-beta-7 integrin) and PF-00547659, an anti-MadCam mAb, are being developed (Lobaton et al. in Aliment Pharmacol Ther 39(6):579–594, 2014). Oral anti-trafficking agents, such as ozanimod, targeting the S1P receptor responsible for the efflux of T-cells from the lymph nodes, have also shown efficacy in patients with ulcerative colitis (UC) (Sandborn et al. in N Engl J Med 374(18):1754–1762, 2016). Oral agents inhibiting cell signaling have been explored successfully in IBD. Tofacitinib, a non-selective oral Janus kinase (JAK) inhibitor, is effective in patients with UC and several other more or less selective Jak1, 2 and 3 inhibitors are being developed for the treatment of CD and UC (Sandborn et al. in N Engl J Med 376(18):1723–1736, 2017; Vermeire et al. in Lancet 389(10066):266–275, 2017; De Vries et al. in J Crohns Colitis 11(7):885–93, 2017). Finally, despite initial disappointing results with systemic administration of mesenchymal stem cells, Alofisel, adipose tissue derived, allogeneic mesenchymal stem cells, locally injected in perianal fistula tracts, induce long-lasting beneficial effects and the drug has been approved in Europe (Panes et al. in Gastroenterology, 2017). In summary, the quest for new treatment options in IBD is very active and justified by the high medical need and unresolved problems patients are facing.

## Anti-IL12/IL23 agents

The non-selective anti-IL12/23 mAB ustekinumab (Stelara©, Janssen) has been tested in four large phase II/III clinical trials in patients with IBD, and has been proven to be efficacious to induce and maintain clinical remission in CD [9–11]. This treatment has been approved before to treat psoriasis and psoriatic arthritis, and is now also approved in Europe and the US to treat patients with Crohn’s disease. The long-term safety in a large prospective cohort is reassuring, but it has to be said that patients with IBD comprised only 3% of that cohort. Most were patients with psoriasis or rheumatologic conditions [12]. The results of a phase III, randomized, double-blind, placebo-controlled multicenter study to evaluate the safety and efficacy of ustekinumab induction and maintenance therapy in subjects with moderate-to-severe UC (UNIFI, NCT02407236) are expected (Table 1).

**Table 1:** Overview of molecules in clinical development for IBD

MOA	CD	UC
Cytokine/chemokine	MED2070/Risankizumab/Guselkumab (anti-IL23p19)	Ustekinumab
('Selective') anti-adhesion molecules		Etrolizumab PF-00547,659 (anti MadCam) AMG 181 Ozanimod Alicaforsen
Barrier/microbiota	Fecal transplantation	
Signaling	Filgotinib, upadacitinib	Tofacitinib
Cell based	Mesenchymal stem cells (Alofisel)	

*MOA* mechanism of action, *Cd* Crohn’s disease, *UC* ulcerative colitis

The role of ustekinumab pharmacokinetics is unclear at this moment, but cohort data suggest that endoscopic healing is related to ustekinumab trough levels [13], which was also observed in a post hoc sub-analysis of the phase III program [14]. In contrast to infliximab, the immunogenic profile of ustekinumab is very limited (2.3% of all 1154 patients included in the UNITI trials developed auto-antibodies against ustekinumab, measured via a drug-tolerant assay) [11]. This might explain why immunomodulators do not seem to influence ustekinumab pharmacokinetics [14].

**Fig. 1:**
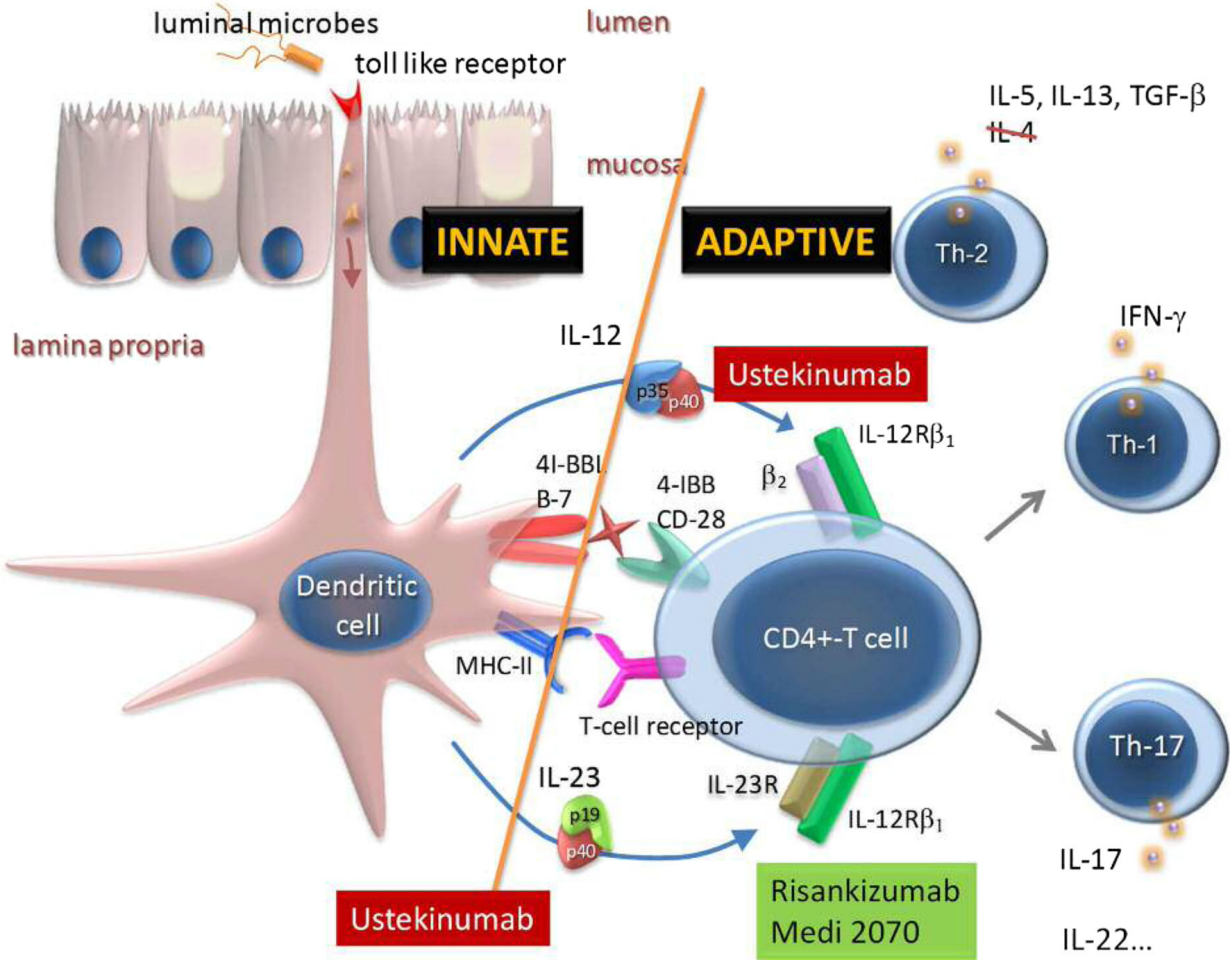
Pro-inflammatory cytokine pathways in IBD

Though the efficacy and safety of blocking p40 has been established, it is not clear if direct modulation of the IL12 axis via p40 contributes to the efficacy or has potential risks related to IL12′s role in tumor immune surveillance and in host defense against intracellular pathogens [2]. Hence, selectively blocking IL23p19 might offer important differentiation in efficacy and safety (Fig. 1).

MEDI2070 (AMG-139, Amgen and MedImmune) is a fully human IgG2 monoclonal antibody, which selectively binds p19. The results of a phase IIa induction study recently demonstrated clinical efficacy in 121 patients with moderate-to-severe CD, who previously had failed anti-TNF therapy [15]. After administration of 700 mg MEDI2070 intravenously at week 0–4, clinical effect (> 100 drop from baseline CDAI-score OR CDAI < 150) at week eight was achieved in 49.2% of patients, compared to 26.7% of patients receiving placebo (*p* = 0.010). Through week 12, no increased rate of adverse events (AE) with active treatment was observed compared to placebo. Similarly to MEDI2070, risankizumab (BI-655066, Boehringer Ingelheim and Abbvie) potently binds to p19 and prevents its binding to the IL23R. The results of a phase II trial in moderately-to-severely active CD were favorable [16]. Selective blockade of IL23p19 with risankizumab was superior to placebo in achieving clinical remission (30.5% vs 15.4% respectively, *p* = 0.049) and clinical response (39.0% vs 20.5% respectively, *p* = 0.027). Ninety-four per cent of all included patients had been exposed to anti-TNF before, with approximately one-third (30%) experiencing primary non-response and another third (28%) secondary loss of response, reflecting a very refractory population. In addition, significantly more patients achieved endoscopic remission with risankizumab compared to placebo (17.1% vs 2.6%, respectively; *p* = 0.002) at week 12. So far, risankizumab shows a favorable safety profile with fewer severe or serious AE reported compared to placebo. Although ustekinumab and risankizumab have not yet been compared head-to-head in IBD, a phase II randomized-trial in patients with psoriasis showed superiority of risankizumab compared to ustekinumab [17].

LY3074828 (Eli Lilly) is actually being studied in patients with moderate-to-severe UC (NCT02589665). Tildrakizumab (MK-3222, Sun Pharma and Merck) will potentially be studied in active CD, after the first positive results of a phase IIa trial in psoriasis [18]. Finally, guselkumab (Janssen Biotech) showed efficacy in a recent phase II trial in psoriasis [19, 20], and early trials in patients with IBD are underway.

Targeting IL17, a key cytokine secreted by T_H_17 cells and downstream mediator of IL23 signaling, is logical as an increased expression of IL17A and IL17F has been reported in active CD, scattered throughout the submucosa and muscularis propria [21, 22]. Brodalumab (AMG827, Amgen) is a fully human antibody against the IL17-receptor A (IL17RA), studied in a phase II trial in moderate-to-severe CD. The study was terminated prematurely after an independent review of unblinded safety data from 117 of 216 planned subjects demonstrated an imbalance in worsening CD in active treatment groups [23]. Secukinumab (AIN457, Novartis) is a fully humanized selective anti-IL17A antibody (Fig. 1), studied in CD after increased expression of IL17A mRNA was reported in the intestinal mucosa of CD patients [24]. Phase I-II trials in psoriasis and rheumatoid arthritis showed clinically relevant responses and a head-to-head comparative trial in patients with plaque psoriasis showed superiority for secukinumab over the anti-p40 ustekinumab [25]. Nevertheless, an RCT in moderate-to-severe CD demonstrated blockade of IL17A was ineffective and secukinumab may even worsen disease in patients with a certain genotype. In addition, higher rates of AE, mainly serious infections (mucocutaneous candidiasis) were noted compared to placebo [26].

The fact that blockade of either the ligand (secukinumab) or its receptor (brodalumab) causes worsening disease, suggests this is not merely coincidence but probably a true biologic effect. The worsening comes not entirely unexpected, as IL17A has been claimed to show both a protective and exacerbating effect in preclinical murine models [2]. Both RCTs clearly point out that blocking IL17/IL17R may interfere with a protective function of IL17 in the intestine.

## Anti-adhesion molecules

**Fig. 2:**
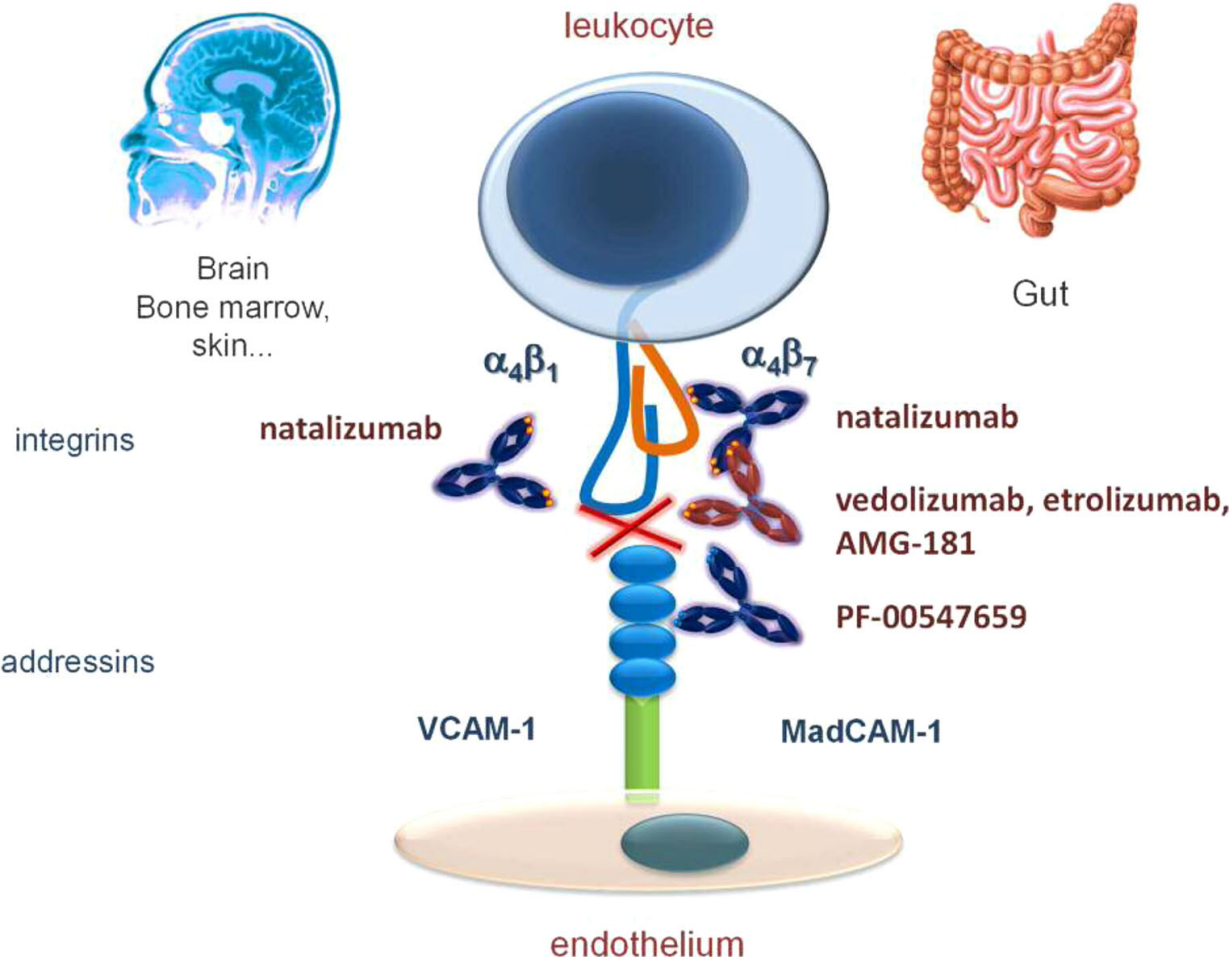
Anti-integrins

Vedolizumab (Entyvio^©^, Takeda), a mAb targeting a4b7 integrins resulting in a gut selective mechanism of action (Fig. 2), has been approved for the treatment of moderate to severe Crohn’s disease and ulcerative colitis worldwide. Natalizumab, a non-selective anti-a4 integrin mAb, had been shown to be effective in Crohn’s disease before, but is only available to treat Crohn’s disease in the US and Switzerland. Other jurisdictions have not approved this drug since it carries a risk of a potentially deadly viral brain disease, progressive multifocal leukencephalopathy [3]. The long-term risk in patients with multiple sclerosis treated long term is estimated at 1/300. With vedolizumab, no cases have been reported so far in over 72,000 exposed patients.

Etrolizumab (Genentech-Roche) is a slightly less gut selective mAb targeting the beta7 integrin subunit and thus both alpha4beta7 and aEbeta7. This could potentially increase its efficacy as aEb7 integrins, through their binding to E-cadherin, are responsible for retention of lymphocytes in the diseased tissue. On the other hand, this broader mechanism of action may result in a loss of gut selectivity and thus in more systemic side-effects. Etrolizumab was proven to be efficacious in a phase IIb trial in moderate to severe ulcerative colitis [27]. The remission rates in the 100 mg and 300 mg SC group combined were significantly better than placebo (10% 300 mg, 21% 100 mg, 0% placebo, *p* = 0.048 and *p* = 0.004 respectively). Of note, etrolizumab at either dose of 100 and 300 mg was not more effective than placebo in patients who had already been treated with anti-TNF agents before. A large phase III trial is currently recruiting patients.

Inhibiting mucosal addressin cellular adhesion molecule 1 (MadCAM-1), the ligand of a4b7 integrin, also is a gut-selective anti-adhesion molecule strategy. The anti-MadCAM-1 m Ab, PF-00547, 659 (Pfizer and Shire) has been tested in both Crohn’s disease and ulcerative colitis, but clear significant improvements over placebo of clinical disease activity have not been shown yet [28].

Fingolimod, an oral anti-adhesion molecule targeting the S1P receptor, is used in clinical practice in patients with multiple sclerosis. The binding of S1P to its receptor, guides lymphocytes out of the lymph nodes and therefore, interfering with this mechanism, results in a sequestration of lymphocytes in the lymph nodes. Ozanimod, an S1P receptor antagonist, has shown efficacy in patients with moderate-to-severe ulcerative colitis. Ozanimod 1 mg, but not the lower dose of 0.5 mg, was significantly superior to placebo (ozanimod 1 mg 16.4%, ozanimod 0.5 mg 13.8%; placebo 6.2%; *p* = 0.048 and *p* = 0.14 respectively) [4]. Both doses were better than placebo at inducing mucosal healing. Other similar molecules are being developed to treat IBD. S1P receptors carry a risk of systemic infections, including JC virus induced brain infections, and bradyarrhythmia, but this risk may vary based on the S1P receptor subtypes targeted by the different compounds.

## Janus kinase inhibitors

The Janus kinase (JAK) enzymes, named after the two-faced Roman god Janus, are crucial in the signaling of a variety of cytokines through their receptor and always occur in heterodimers. Different combinations of JAK 1, 2, 3 and Tyrosine kinase (TYK) 2 are involved in the signaling of key inflammatory cytokines. The specificity of a molecule for the different JAK subtypes therefore will determine its efficacy and safety profile. The non-selective JAK inhibitor tofacitinib (Pfizer) is approved in Europe and other parts of the world for the treatment of rheumatoid arthritis. Also, in moderate to severe ulcerative colitis, tofacitinib at a dose of 3 to 15 mg BID is more effective than placebo to induce clinical remission [5]. The results of two large phase III trials (Octave 1 and 2) confirm the efficacy at inducing remission in ulcerative colitis, and the results of the maintenance phase of these trials indicate that tofacitinib is also effective at maintaining remission throughout one year [5]. On the other hand, tofacitinib failed to show clinical efficacy in Crohn’s disease [29]. Other compounds, such as the more JAK1 selective filgotinib (Galapagos/Gilead) and upadacitinib (Abbvie) are being developed to treat Crohn’s disease and ulcerative colitis. Filgotinib is more effective than placebo to induce clinical remission and mucosal healing in patients with moderate to severe Crohn’s disease [6]. Results from a phase II RCT with upadacitinb are also showing dose dependent favorable outcomes in patients with Crohn’s disease [30]. JAKinibs are associated with an increase in herpes zoster infections and potentially with other systemic infections, serum lipid disturbances and anemia [7]. Ongoing phase III trials may elucidate whether the safety profile is determined by the selectivity of compounds for JAK1,2,3 and Tyk2 respectively.

## Fecal microbiota transplantation

Treating IBD with fecal material has been tried for more than 2000 years. All data available until recently, were uncontrolled [31]. However, the renewed interest in the intestinal microbiome as a modifier of human disease, has led to randomized controlled trials using fecal transplantation in patients with ulcerative colitis. Most of the fecal mass is comprised of microbiota, and therefore the term fecal microbiota transplantation (FMT) has been used. In total, 3 out of the 4 RCTs with FMT which have been performed in recent years, show a significant and favourable effect in inducing clinical and/or endoscopic remission in patients with UC [32–35]. Nevertheless, more research is needed on the ideal microbiome composition and FMT conditions, such as mode and intensity of administration, to treat UC and CD.

## Nucleotides

The oral anti-sense small oligonucleotide Mongersen (Guliani/Cellgene) is directed against the translation of SMAD7. This is a key inhibitory protein that downregulates the signaling of Transforming growth factor-beta (TGF-b). When SMAD7 protein is suppressed, TGF-b will be able to resort its anti-inflammatory effects on the mucosa. The first RCT with Mongersen indicated that this molecule is efficacious to induce clinical remission in patients with Crohn’s disease. Mongersen at the higher doses of 40 and 160 mg given daily for 14 days was better than placebo at inducing Crohn’s disease remission [36]. In addition, Mongersen induced a long-lasting response off therapy [37]. However, a confirmatory randomized control trial was stopped prematurely because of lack of efficacy and the further development of this drug has been halted.

## Mesenchymal stem cells

Stem cell therapy has not been successful in IBD until the advent of mesenchymal stem cell therapy to treat perianal Crohn’s disease. Cx-601, Alofisel (Tigenix/Takeda) has proven to be efficacious to induce and maintain fistula closure, when applied locally close to the tract in conjunction with surgical preparation of the fistula track [8]. Of note, a high placebo effect was noted in this trial, which could have been due to the background therapies including anti-TNFs and the surgical preparation of the fistula track with closing of the internal orifice in both treatment arms. The drug received approval in Europe and a second phase III trial is being conducted.

## Summary

The landscape of IBD treatment is widening rapidly. As more biologic and small molecule therapies become available, patients and clinicians alike will be faced with selecting the right drug. The mechanisms of action and perceived tolerability of new treatment options will increasingly drive clinical decisions. Head-to-head comparative trials are desperately needed to facilitate these important choices.
